# Case Report: A case of *Salmonella* spondylitis masquerading as tuberculosis in a child

**DOI:** 10.3389/fmed.2025.1754318

**Published:** 2026-01-23

**Authors:** Shuai Guo, Yu Zhu

**Affiliations:** 1 Department of Pediatrics, West China Second University Hospital of Sichuan University, Chengdu, China; 2 Key Laboratory of Birth Defects and Related Diseases of Women and Children, Ministry of Education, Chengdu, China

**Keywords:** abscess, children, osteomyelitis, *Salmonella*, spondylitis

## Abstract

**Background:**

*Salmonella* typically causes gastroenteritis and rarely leads to invasive infections.

**Case presentation:**

A 14-year-old boy, without a definitive history of an unsanitary diet or open wounds, was residing in an area with a high prevalence of tuberculosis. His primary symptoms included fever, cough, lumbar pain, and weight loss. The initial pathogen test was negative. Medical imaging revealed pulmonary nodules, intervertebral space narrowing, vertebral bone destruction, and a psoas muscle abscess. Empirical antibiotic therapy and diagnostic anti-tuberculosis treatment yielded poor results. Ultimately, pathogen testing of the surgically excised lesion identified *Salmonella Dublin*. Antimicrobial therapy guided by susceptibility testing yielded favorable outcomes.

**Conclusion:**

Empirical therapy is often necessary during the initial phase of treatment. However, clinicians should consider uncommon conditions and employ appropriate approaches to obtain pathogen-specific test results, which can guide targeted therapeutic strategies when the anticipated clinical outcome is suboptimal.

## Background

*Salmonella* is one of the important causes of human foodborne disease. Non-typhoidal *Salmonella* refers to serotypes other than *Salmonella typhi* and *Salmonella paratyphi*. These non-typhoidal serotypes can cause a variety of clinical infections in humans. Most cases of non-typhoidal *Salmonella* infections typically present as gastroenteritis. However, in some patients, non-typhoidal *Salmonella* can cause invasive infections, including bacteremia, meningitis, and osteomyelitis (1). The incidence of invasive non-typhoidal *Salmonella* infections has been estimated at 44.8 per 100,000 persons per year (2). Here, we present a case of spondylitis caused by *Salmonella Dublin*, highlighting its challenging diagnostic and therapeutic course.

## Case presentation

A 14-year-old boy was admitted to the pediatric infectious disease department due to “16 days of waist pain and 10 days of fever,” accompanied only by a mild cough. The patient received all standard immunizations, including hepatitis B vaccine, bacille Calmette-Guerin (BCG) vaccine, diphtheria-tetanus-pertussis vaccine, and polio vaccine. He had a history of recurrent atopic dermatitis in early childhood and no history of animal contact, a definitive unsanitary diet, travel to epidemic areas, or inherited disease. From the onset of symptoms to admission, he had lost 4.5 kg.

Physical examination revealed tenderness in the left upper quadrant and thoracolumbar spine. Laboratory tests showed the following: Complete blood count (CBC): white blood cell (WBC) 5.3 × 10^9/L, neutrophil (N) 73.2%, hemoglobin (HGB) 134 g/L, and platelet (PLT) 237 × 10^9/L; C-reactive protein (CRP) 41.9 mg/L; and erythrocyte sedimentation rate (ESR) 38 mm/h. He received empirical treatment with cefoperazone/sulbactam, but there was no improvement. We adjusted the antibiotic regimen from targeting Gram-positive resistant bacteria to include Gram-negative resistant bacteria. After administering vancomycin combined with meropenem for five days, his temperature returned to normal.

**Figure 1:**
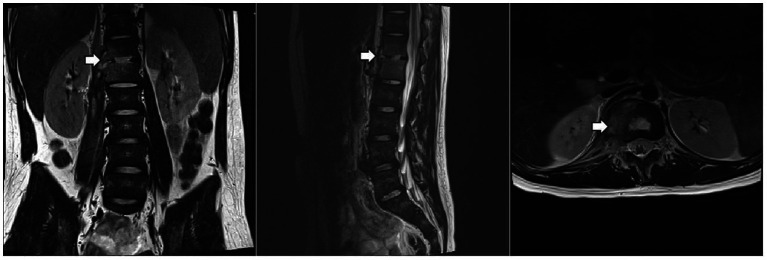
Thoracolumbar MRI. The arrow indicates localized bone destruction of the T12 and L1 vertebral bodies, narrowing of the intervertebral spaces, and abnormal signals within the intervertebral discs.

The patient underwent a series of tests to identify the cause of his illness, all of which were negative. These included urine and blood cultures, Epstein–Barr virus (EBV) DNA and antibody tests, serum 1,3-*β*-D-glucan and galactomannan tests, tuberculin skin test (TST), interferon-gamma release assay (IGRA), *cryptococcus neoformans* antigen test, serologic markers for hepatitis B, HIV antibody test, *Treponema Pallidum* antibody test, blood metagenomic next-generation sequencing (mNGS), and bone marrow culture. His serum immunoglobulin levels were normal, as were the counts and percentages of B, NK, and T lymphocytes. Computed tomography of the chest and abdomen showed pulmonary nodules, liver and spleen enlargement, intervertebral space narrowing from T12 to L1 with nodules, and local compression of the adjacent vertebral body. Thoracolumbar magnetic resonance imaging (MRI) (Figure 1) showed bone marrow edema of T12-L1, local bone destruction of T12 and L1, narrowing of the corresponding intervertebral space, abnormal signals in the intervertebral disc, paraspinal soft tissue swelling, and a right psoas muscle abscess.

Tuberculosis was initially suspected based on imaging findings, symptoms (fever, cough, and weight loss), and epidemiological history (residence in an area with a high prevalence of tuberculosis). He received diagnostic anti-tuberculosis treatment with isoniazid, rifampin, pyrazinamide, and ethambutol (HRZE) after obtaining the consent of the guardian. He also underwent a bronchoscopy with bronchoalveolar lavage. The lavage fluid tested negative for acid-fast staining, *Mycobacterium tuberculosis* culture, and mNGS (tuberculosis).

**Figure 2:**
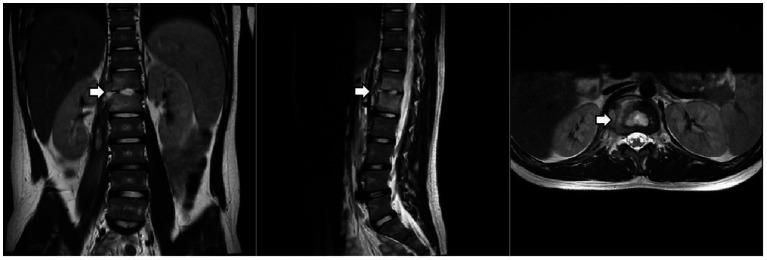
Thoracolumbar MRI. The arrow indicates that the lesion at the T12-L1 level had expanded.

Given the predominance of Gram-positive bacteria in osteoarticular infections, meropenem was discontinued first, after his temperature had remained normal for one week. After two weeks of anti-tuberculosis therapy, repeat thoracolumbar MRI (Figure 2) showed progression of the T12 and L1 lesions. He underwent surgical debridement, and the excised tissue was sent for pathological examination and pathogen identification. The patient developed a fever again after surgery. Laboratory tests showed the following: CBC: WBC 8.1 × 10^9/L, N% 76.4, HGB 125 g/L, and PLT 304 × 10^9/L; CRP 77.9 mg/L. Imipenem/cilastatin was added to cover resistant Gram-negative bacteria.

**Figure 3:**
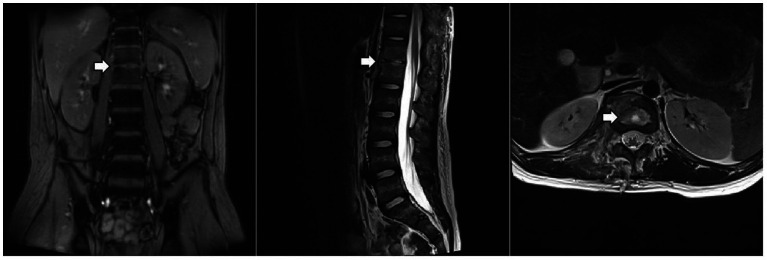
Thoracolumbar MRI. The arrow indicates that the lesion at the T12-L1 level had improved.

**Figure 4:**
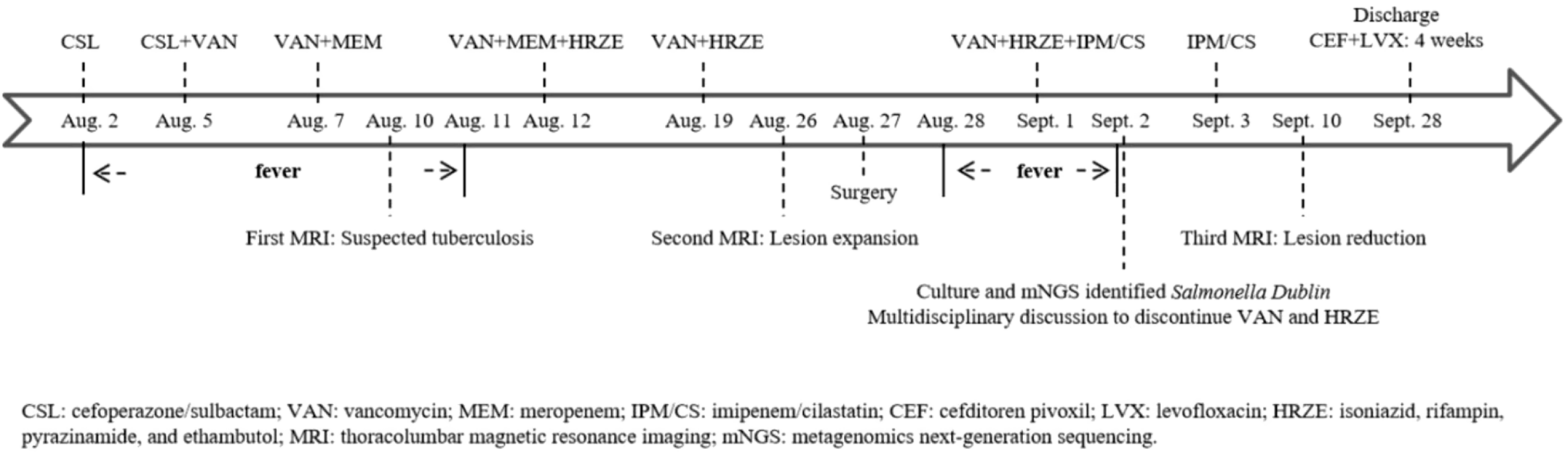
Timeline of the patient’s treatment.

Culture and metagenomic next-generation sequencing (mNGS) of the surgically excised lesion identified *Salmonella Dublin*. Through multidisciplinary discussion involving the pediatric infectious diseases department, orthopedics department, radiology department, and pharmacy department, we concluded that the patient’s imaging findings were consistent with invasive non-typhoidal *Salmonella* infection and that the pathogen identified by both culture and mNGS was highly credible. Treatment with imipenem/cilastatin was continued according to antimicrobial sensitivity tests, while HRZE and vancomycin were discontinued. After two days of imipenem/cilastatin therapy, the patient had no further episodes of fever. After 10 days of imipenem/cilastatin therapy, follow-up thoracolumbar MRI (Figure 3) showed a reduction in bone marrow edema at T12-L1, as well as decreased lesions in the paraspinal soft tissue and the right psoas muscle. The patient was discharged after completing four weeks of treatment with imipenem/cilastatin and subsequently continued oral therapy with cefditoren pivoxil and levofloxacin for an additional four weeks. The patient attended follow-up visits at a local hospital. We learned from a phone call that he had recovered and resumed his studies (Timeline of the patient’s treatment see Figure 4).

## Discussion and conclusion

Non-typhoidal *Salmonella* infections involving the bloodstream or other normally sterile sites are referred to as invasive infections, and several host factors are associated with their development. Age is a common risk factor for the development of invasive infections. In a global burden survey of invasive non-typhoidal *Salmonella* disease, children (<5 years) and older adults (≥70 years) represented the two peaks in the age distribution (3). This patient was not in a high-risk age group and did not have gastrointestinal symptoms, so non-typhoidal *Salmonella* infection was not considered initially.

Non-typhoidal *Salmonella* is transmitted via the fecal-oral route. Humans may become infected through the consumption of contaminated milk, eggs, meat, or fresh produce or through contact with infected animals (4–8). We repeatedly inquired about the patient’s epidemiological history but failed to identify a definitive source of infection. However, we suspect gastrointestinal transmission as the route of infection, given the patient’s consumption of potentially contaminated food. In addition, some patients with non-typhoidal *Salmonella* infection continue to shed the bacteria for an extended period of time (months or even years) (9). The patient may have acquired the infection through person-to-person transmission while living in a school dormitory (10).

Primary immunodeficiency diseases, such as chronic granulomatous disease (CGD) and Mendelian susceptibility to mycobacterial disease (MSMD), also increase host susceptibility to invasive non-typhoidal *Salmonella* infections. CGD is a primary immunodeficiency disease of phagocytes—including neutrophils, monocytes, macrophages, and eosinophils—caused by mutations in NADPH oxidase. This defect limits superoxide production, impairing the ability of phagocytes to kill pathogens intracellularly and leading to increased risk of infection dissemination (11). Patients with MSMD are susceptible to non-typhoidal *Salmonella* infection due to a primary immunodeficiency affecting the IL-12/IL-23-IFNγ pathway (12). Many studies have shown the importance of IFNγ-mediated immunity in the host control of non-typhoidal *Salmonella* infections (13–17). In addition, patients with diabetes mellitus, chronic corticosteroid use, hematologic neoplasms, autoimmune diseases, organ transplants, or immunosuppressive drug use are also susceptible to invasive non-typhoidal *Salmonella* infections (18–23). The patient did not have any of the aforementioned conditions, and genetic testing for immunodeficiency disease did not reveal any known pathogenic mutations (Supplementary Table 1).

The diagnostic and therapeutic course in this case was particularly challenging. Initially, clinical evidence strongly suggested a tuberculosis infection. However, anti-tuberculosis treatment failed to improve the condition. To establish a definitive diagnosis, surgical debridement was performed to obtain tissue samples for pathogen identification. Subsequent culture and mNGS conclusively identified *Salmonella* as the causative agent. Fortunately, guided by antimicrobial susceptibility testing, targeted antibiotic therapy achieved favorable clinical outcomes. Previous reports of similar cases have shown that bone and joint infections caused by non-typhoidal *Salmonella* are often difficult to diagnose in the early stages, with the pathogen frequently being identified only after surgical debridement (24–26).

In conclusion, accurate identification of the causative pathogen is critical for effective treatment. When diagnostic treatment proves ineffective, the possibility of a rare pathogen or an atypical pathogen should be considered, and appropriate clinical specimens should be collected to confirm the pathogen.

## Data Availability

The original contributions presented in the study are included in the article/Supplementary material, further inquiries can be directed to the corresponding author.
