# Supplementary material for: Case Report: A case of Salmonella spondylitis masquerading as tuberculosis in a child
Source: Front Med (Lausanne). 2026 Jan 23;12:1754318. doi: 10.3389/fmed.2025.1754318 (PMC12875919; doi:10.3389/fmed.2025.1754318)
Supplement: Supplementary file 1 [file Data_Sheet_1.pdf]

Supplement 1. Genetic testing list

ACD	*ACP5*	*ACTB*	*ADA*	*ADA2*	*ADAM17*	*ADAR*	*ADGRE2*
AICDA	*AIRE*	*AK2*	*ALPI*	*AP1S3*	*AP3B1*	*AP3D1*	*APOL1*
ARHGEF1	*ARPC1B*	*ATM*	*ATP6AP1*	*B2M*	*BACH2*	*BCL10*	*BCL11B*
BLM	*BLNK*	*BRCA1*	*BRCA2*	*BRIP1*	*BTK*	*C1QA*	*C1QB*
C1QC	*C1R*	*C1S*	*C2*	*C3*	*C4A*	*C4B*	*C5*
C6	*C7*	*C8A*	*C8B*	*C8G*	*C9*	*CARD11*	*CARD14*
CARD9	*CARMIL2*	*CASP10*	*CASP8*	*CCBE1*	*CD19*	*CD247*	*CD27*
CD3D	*CD3E*	*CD3G*	*CD40*	*CD40LG*	*CD46*	*CD55*	*CD59*
CD70	*CD79A*	*CD79B*	*CD81*	*CD8A*	*CDCA7*	*CEBPE*	*CFB*
CFD	*CFH*	*CFHR1*	*CFHR2*	*CFHR3*	*CFHR4*	*CFHR5*	*CFI*
CFP	*CFTR*	*CHD7*	*CIB1*	*CIITA*	*CLCN7*	*CLEC7A*	*CLPB*
COL7A1	*COLEC11*	*COPA*	*CORO1A*	*CR2*	*CSF2*	*CSF2RA*	*CSF2RB*
CSF3R	*CTC1*	*CTLA4*	*CTPS1*	*CTSC*	*CXCR4*	*CYBA*	*CYBB*
CYBC1	*DBR1*	*DCLRE1B*	*DCLRE1C*	*DDX58*	*DEF6*	*DKC1*	*DNAJC21*
DNASE1L3	*DNASE2*	*DNMT3B*	*DOCK2*	*DOCK8*	*EFL1*	*ELANE*	*EPG5*
ERBIN	*ERCC4*	*ERCC6L2*	*EXTL3*	*FAAP24*	*FADD*	*FANCA*	*FANCB*
FANCC	*FANCD2*	*FANCE*	*FANCF*	*FANCG*	*FANCI*	*FANCL*	*FANCM*
FAS	*FASLG*	*FAT4*	*FCGR3A*	*FCHO1*	*FCN3*	*FERMT1*	*FERMT3*
FOXN1	*FOXP3*	*FPR1*	*G6PC3*	*G6PD*	*GATA2*	*GFI1*	*GINS1*
GUCY2C	*HAVCR2*	*HAX1*	*HELLS*	*HMOX1*	*HPS1*	*HPS4*	*HPS6*
HYOU1	*ICOS*	*ICOSLG*	*IFIH1*	*IFNAR1*	*IFNAR2*	*IFNG*	*IFNGR1*
IFNGR2	*IGHM*	*IGKC*	*IGLL1*	*IKBKB*	*IKBKG*	*IKZF1*	*IL10*
IL10RA	*IL10RB*	*IL12B*	*IL12RB1*	*IL12RB2*	*IL17F*	*IL17RA*	*IL17RC*
IL18BP	*IL1RN*	*IL21*	*IL21R*	*IL23R*	*IL2RA*	*IL2RB*	*IL2RG*
IL36RN	*IL6*	*IL6R*	*IL6ST*	*IL7R*	*INO80*	*IRAK1*	*IRAK4*
IRF2BP2	*IRF3*	*IRF4*	*IRF7*	*IRF8*	*IRF9*	*ISG15*	*ITCH*
ITGB2	*ITK*	*JAGN1*	*JAK1*	*JAK3*	*KDM6A*	*KMT2A*	*KMT2D*
KRAS	*LACC1*	*LAMTOR2*	*LAT*	*LCK*	*LIG1*	*LIG4*	*LPIN2*
LRBA	*LYST*	*MAD2L2*	*MAGT1*	*MALT1*	*MAP3K14*	*MASP1*	*MASP2*

MCM4	MEFV	MOGS	MRE11	MRTFA	MS4A1	MSH6	MSN
MTHFD1	MVK	MYD88	MYSM1	NBAS	NBN	NCF1	NCF2
NCF4	NCSTN	NFAT5	NFE2L2	NFKB1	NFKB2	NFKBIA	NHEJ1
NHP2	NLRC4	NLRP1	NLRP12	NLRP3	NOD2	NOP10	NRAS
NSMCE3	OAS1	ORAI1	OSTM1	OTULIN	PALB2	PARN	PEPD
PGM3	PIK3CD	PIK3R1	PLCG2	PLEKHM1	PMS2	PNP	POLA1
POLD1	POLD2	POLE	POLE2	POLR3A	POLR3C	POLR3F	PRF1
PRKCD	PRKDC	PSEN1	PSENEN	PSMB8	PSMG2	PSTPIP1	PTEN
PTPRC	RAB27A	RAC2	RAD51	RAD51C	RAG1	RAG2	RANBP2
RASGRP1	RBCK1	REL	RELA	RELB	RFWD3	RFX5	RFXANK
RFXAP	RHOH	RIPK1	RMRP	RNASEH2A	RNASEH2B	RNASEH2C	RNF168
RNF31	RNU4ATAC	RORC	RPSA	RTEL1	SAMD9	SAMD9L	SAMHD1
SBDS	SEC61A1	SEMA3E	SERPING1	SH2D1A	SH3BP2	SH3KBP1	SHARPIN
SKIV2L	SLC29A3	SLC35C1	SLC37A4	SLC39A7	SLC46A1	SLC7A7	SLX4
SMARCAL1	SMARCD2	SNX10	SP110	SPINK5	SPPL2A	SRP54	SRP72
STAT1	STAT2	STAT3	STAT5B	STIM1	STK4	STN1	STX11
STXBP2	TAP1	TAP2	TAPBP	TAZ	TBK1	TBX1	TCF3
TCIRG1	TCN2	TERC	TERT	TFRC	TGFB1	TGFBR1	TGFBR2
THBD	TICAM1	TINF2	TIRAP	TLR3	TMC6	TMC8	TMEM173
TNFAIP3	TNFRSF11A	TNFRSF13B	TNFRSF13C	TNFRSF1A	TNFRSF4	TNFRSF9	TNFSF11
TNFSF12	TOP2B	TP53	TPP1	TPP2	TRAC	TRAF3	TRAF3IP2
TREX1	TRIM22	TRNT1	TTC37	TTC7A	TYK2	UBE2T	UNC13D
UNC93B1	UNG	USB1	USP18	VPS13B	VPS45	WAS	WDR1
WIPF1	WRAP53	XIAP	XRCC2	ZAP70	ZBTB24	ZNF341	
